# Regional and sub-regional differences in hippocampal GABAergic neuronal vulnerability in the TgCRND8 mouse model of Alzheimer's disease

**DOI:** 10.3389/fnagi.2015.00030

**Published:** 2015-03-24

**Authors:** Marilia S. Albuquerque, Ian Mahar, Maria Antonietta Davoli, Jean-Guy Chabot, Naguib Mechawar, Rémi Quirion, Slavica Krantic

**Affiliations:** ^1^Douglas Mental Health University InstituteVerdun, QC, Canada; ^2^Laboratory of Biomedicine and Biotechnology, School of Arts, Sciences and Humanities, Universidade de São PauloSão Paulo, Brazil; ^3^Graduation Course on Pharmacology, Institute of Biomedical Sciences, Universidade de São PauloSão Paulo, Brazil; ^4^Research Group on Neuropharmacology of AgingSão Paulo, Brazil; ^5^McGill Group for Suicide Studies, Douglas Mental Health University InstituteVerdun, QC, Canada; ^6^Integrated Program in Neuroscience, McGill UniversityMontreal, QC, Canada; ^7^Department of Psychiatry, McGill UniversityMontreal, QC, Canada; ^8^Centre de Recherche des Cordeliers, INSERM, Université Paris Descartes, Sorbonne Paris Cité, UMR_S 1138, Université Pierre et Marie Curie Univ Paris 06, Sorbonne UniversitésParis, France

**Keywords:** Alzheimer's disease, somatostatin, neuropeptide Y, parvalbumin, hippocampal sub-regions

## Abstract

Hippocampal network activity is predominantly coordinated by γ-amino-butyric acid (GABA)ergic neurons. We have previously hypothesized that the altered excitability of hippocampal neurons in Alzheimer's disease (AD), which manifests as increased *in vivo* susceptibility to seizures in the TgCRND8 mouse model of AD, may be related to disruption of hippocampal GABAergic neurons. In agreement, our previous study in TgCRND8 mice has shown that hippocampal GABAergic neurons are more vulnerable to AD-related neuropathology than other types of neurons. To further explore the mechanisms behind the observed decrease of GABAergic neurons in 6 month-old TgCRND8 mice, we assessed the relative proportion of somatostatin (SOM), neuropeptide Y (NPY) and paravalbumin (PV) sub-types of GABAergic neurons at the regional and sub-regional level of the hippocampus. We found that NPY expressing GABAergic neurons were the most affected, as they were decreased in CA1-CA2 (pyramidal-, stratum oriens, stratum radiatum and molecular layers), CA3 (specifically in the stratum oriens) and dentate gyrus (specifically in the polymorphic layer) in TgCRND8 mice as compared to non-transgenic controls. SOM expressing GABAergic neurons were decreased in CA1-CA2 (specifically in the stratum oriens) and in the stratum radiatum of CA3, whereas PV neurons were significantly altered in stratum oriens sub-region of CA3. Taken together, these data provide new evidence for the relevance of hippocampal GABAergic neuronal network disruption as a mechanism underlying AD sequelae such as aberrant neuronal excitability, and further point to complex hippocampal regional and sub-regional variation in susceptibility to AD-related neuronal loss.

## Introduction

Alzheimer's disease (AD) is an age-related neurodegenerative disorder characterized by progressive loss of cognitive and executive functions. The main histological hallmarks of AD are the extracellular deposition of amyloid beta (Aβ) peptides into the extracellular amyloid-plaques and the intracellular accumulation of hyperphosphorylated tau protein (neurofibrillary tangles) (Hardy and Selkoe, 2002; Hardy, 2006). Clinically, the disease manifests first by cognitive complaints at a stage considered as a prodromal (silent) phase of AD, also known as MCI (mild cognitive impairment). For the reasons that remain unknown, not all of subjects with MCI will convert to AD (Nelson and O'Connor, 2008) with a “probable” clinical diagnosis based mainly on PET-scan imaging of Aβ accumulation into amyloid-plaques and elevated Aβ 42/Aβ 40 ratio in cerebrospinal fluid. At the time of diagnosis, significant hippocampal atrophy is already detectable in some patients. As AD progresses, the neuronal loss increases, yielding a significant neurodegeneration which is generally observed at the terminal stages in post-mortem studies (McKhann et al., 1984).

We and others have previously reported a loss of hippocampal GABAergic neurons in 6 month-old mice in AD models such as AβPPdE9 (Ramos et al., 2006) and TgCRND8 (Krantic et al., 2012). Remarkably, the TgCRND8 mice used in our studies display synaptic hyperexcitability in the CA1 hippocampal region, which is detectable at 5 months (Jolas et al., 2002). Moreover, *in vivo* treatment of 1.5–2 month-old TgCRND8 mice with pentylenetetrazole, a drug acting on the picrotoxin site of type-A GABA receptors, has revealed latent GABA-related hippocampal network impairments (Del Vecchio et al., 2004). These findings strongly support the recently-proposed hypothesis that epileptiform activity is the cause, rather than a consequence, of neurodegeneration in AD (Palop et al., 2007; Gleichmann et al., 2011). According to this hypothesis, the Aβ-mediated increase in hippocampal network excitability is associated with increased global excitatory activity, with subsequent negative impact on learning and memory (Palop et al., 2007; Palop and Mucke, 2009).

Phenotypic analysis of hippocampal GABAergic neuronal populations at the overt stages of AD has pointed to selective loss in number or function of their specific sub-types, notably neurons expressing somatostatin (SOM), neuropeptide Y (NPY) (Ramos et al., 2006) and paravalbumin (PV) (Verret et al., 2012). These studies have reported some regional selectivity of SOM neuronal loss, with stratum oriens in CA- and hilar perforant path-associated NPY neurons in the dentate gyrus (DG) being the most vulnerable (Ramos et al., 2006). However, the putative sub-regional differences regarding respective layers of SOM and NPY vulnerability to Aβ have not been explored in detail. These GABAergic neurons in CA stratum oriens and DG hilus are centrally involved in the innervation of the distal dendritic arbor of pyramidal and granular neurons (Maccaferri and Lacaille, 2003) and fire at theta (4–10 Hz) frequency (Klausberger et al., 2003, 2004), suggesting their involvement in generation of endogenous theta oscilations. In addition to CA1, CA3, and DG, the subiculum also contains an endogenous generator of theta oscillatory activity (Goutagny et al., 2009; Jackson et al., 2014). In contrast to CA1 and CA3, where low (approximately 40–80 Hz) and high (around 120–160 Hz) gamma oscillations are not found intrinsically, these are both generated in the subiculum (Goutagny et al., 2009). Given that gamma oscillations are critically controlled by GABAergic interneurons, that they are embedded into theta rhythm, and that this gamma-theta coupling is crucial for learning and memory (Goutagny et al., 2009), the putative alterations of SOM and NPY GABAergic neurons in the subiculum may be critical in AD. In this study, we therefore asked whether SOM-, NPY-, and PV-expressing GABAergic neurons are selectively lost in specific regions (CA1/CA2, CA3, dentate gyrus, and subiculum) and sub-regions (respective layers) of the hippocampus in 6 month-old TgCRND8 mouse. This age was chosen based on our previous study showing that the overall population of GABAergic neurons in TgCRND8 mice is first decreased in hippocampal CA regions at this age (Krantic et al., 2012).

## Materials and methods

### Animals

TgCRND8 mice bear Swedish KM670/671NL and Indiana V717F mutations in the Aβ precursor encoding gene. By 3–4 months, they overexpress human Aβ, encoded by a double mutated form of hAβAPP (695 Swedish KM670/671NL and Indiana V717F APP mutations) transgene. Memory deficits and Aβ pathology progress during the course of aging, and 6 month-old TgCRND8 mice have a high Aβ level and severe plaque load in many brain regions, including the hippocampus (Chishti et al., 2001).

All experiments followed the policies and guidelines of the Canadian Council on Animal Care, the Animal Care regulations of the McGill University, guidelines on the use of laboratory animals and the U.S. National Institutes of Health Guide for the Care and Use of Laboratory Animals. Male TgCRND8 and wild-type (WT) mice were maintained on an outbred C3H/C57BL6 background and kept on a 12 h light/dark cycle with food and water *ad libitum*.

### Immunohistochemistry on free-floating sections

Animals (*n* = 3 per experimental group) were transcardially perfused (PBS followed by 4% PFA), and brains were stored in fixative for 24 h at 4°C, then in a sucrose solution (30% in PBS) for 3 days at 4°C, frozen using dimethylbutane and stored at −80°C. Brains were sliced coronally using a freezing microtome at 40 μm, and free-floating sections were stored in a cryopreservative solution (3:3:4 glycerol:ethylene glycol:PBS) at −20°C in preparation for immunohistochemistry (IHC) staining. Sections (serial sectioning fraction = 1/8) were incubated in PBS with 0.2% Triton X–100 for 1.5 h, followed by 3% H_2_O_2_ prepared in PBS for 10 min. The sections were blocked with 2% normal serum in PBS + 0.2% Triton X–100 for 1 h. Primary antibodies (Millipore; SOM: rabbit, 1:1000, Millipore; NPY: rabbit, 1:2000, Abcam; PV: rabbit, 1:5000, Abcam) were applied overnight at 4°C. The immunolabeled product was visualized by using the avidin-biotin complex method (Vectastain elite ABC Kit, Vector Laboratories; 30 min), with IgG conjugate (1 h), followed by DAB (for SOM) or SG (for NPY and PV) chromogen development (DAKO). The stained sections were mounted on slides (dried overnight), dehydrated in ethanol, cleared in xylene, and coverslipped.

### Cell quantification

Immunoreactive (IR) cell somata were counted in each hippocampal region and subregion (from bregma −1.06 mm to bregma −3.88 mm, Franklin and Paxinos, 2007), on a Nikon Eclipse E600 (Kanagawa, Japan) with a 20X objective, by an experimenter blind to group identity. Anatomical regions were determined as per Franklin and Paxinos (2007). CA1 and CA2 were combined (CA1/2) due to the relatively amorphous boundary separating these two regions. Intrasubject counts by an additional experimenter (on a Leica DM 2500 microscope) of an additional marker to confirm count/total accuracy correlated significantly (*p* = 0.0062), with Pearson r and intraclass correlation values >0.5. Quantification is shown as cells per section. Pairwise comparisons between WT and Tg animals in hippocampal regions and subregions were performed using unpaired *t*-tests, with Welch's correction applied when required due to data variance. *P* < 0.05 were considered significant.

## Results

### Regional and sub-regional distribution of NPY-expressing neurons

**Figure 1:**
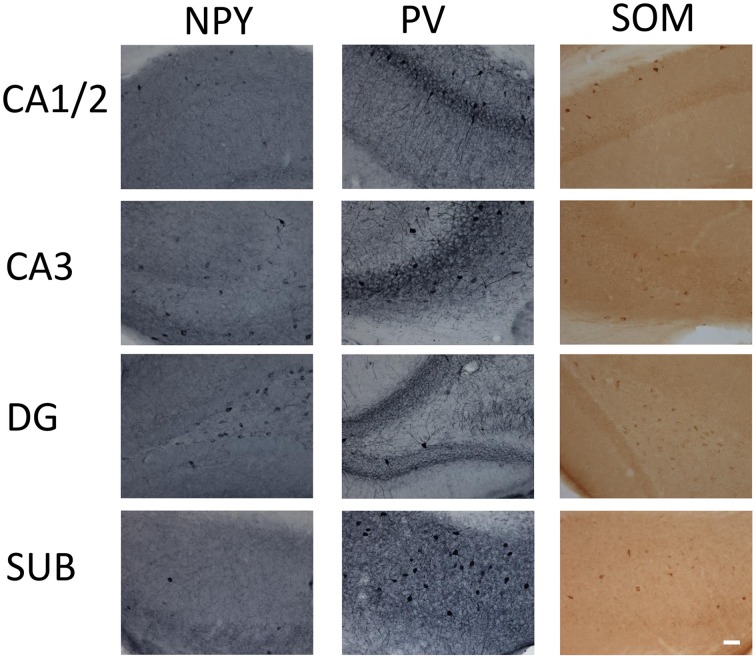
**Micrographs of immunohistochemical labeling of interneurons for parvalbumin (PV), neuropeptide Y (NPY), and somatostatin (SOM) in the CA1 and 2, CA3, dentate gyrus (DG), and subiculum (SUB) hippocampal regions**. Scale bar = 50 μm.

**Figure 2:**
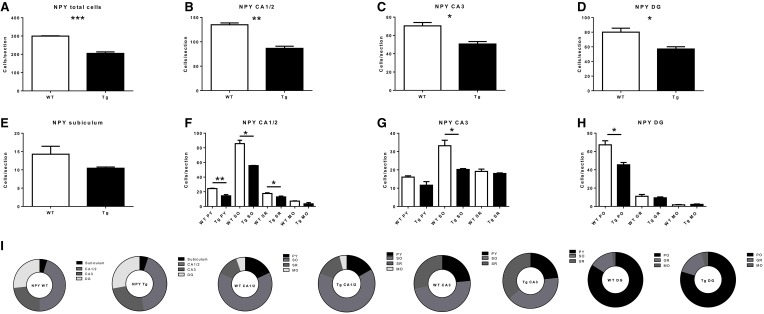
**Quantification of neuropeptide Y- (NPY)-immunoreactive (IR) cells in the overall hippocampus (A), CA1 and CA2 (B), CA3 (C), dentate gyrus (DG; D) and subiculum (E), and in subregions within CA1 and CA2 (F), CA3 (G), and DG, (H)**. **(I)** subregional proportion of NPY-IR cells within hippocampal regions, for wild-type (WT) and transgenic (Tg) animals. PY, pyramidal layer; SO, stratum oriens; SR, stratum radiatum; MO, molecular layer; PO, polymorphic layer; GR, granule cell layer. WT, wild-type; Tg, transgenic. ^*^p < 0.05; ^**^p < 0.01; ^***^p < 0.001.

NPY-IR cells were observed in all CA regions (CA1/CA2, CA3), the DG, and the subiculum of the hippocampal formation of 6 month-old TgCRND8 mice (Figure 1; relative proportions are shown in Figure 2I). This immunoreactivity was very similar, as per visual inspection, to those observed in anatomically matched hippocampal sections of the WT littermates. In contrast, quantification of NPY-IR neurons in the hippocampus as a whole indicated a considerable decrease in their total number in TgCRND8 mice compared to aged-matched WT controls (*p* = 0.0004; Figure 2A). This loss appeared to be distributed throughout hippocampal regions, including CA1/2 (*p* = 0.0011; Figure 2B), CA3 (*p* = 0.012; Figure 2C), and DG (*p* = 0.022; Figure 2D), but not subiculum (*p* = 0.22; Figure 2E).

Sub-regional analyses revealed that the CA1/2 was affected throughout the region. Indeed, the pyramidal layer (*p* = 0.0045), stratum oriens (*p* = 0.022), and stratum radiatum (*p* = 0.037) (but not the molecular layer; *p* = 0.095) displayed a decreased number of NPY-IR neurons in TgCRND8 mice as compared to controls (Figure 2F). The loss of NPY-IR neurons in the CA3 region likely reflects the loss of these neurons in the stratum oriens layer (*p* = 0.013; Figure 2G), as the pyramidal layer (*p* = 0.10) and stratum radiatum (*p* = 0.42) were not significantly affected.

In the DG, the decrease in the number of NPY-IR neurons in TgCRND8 mice may be attributed to a selective loss in the polymorphic layer (*p* = 0.013), as the granular (*p* = 0.49) and molecular (*p* = 0.50) DG layers were not significantly affected in Tg mice compared to WT controls (Figure 2H).

### Regional and sub-regional distribution of PV- and SOM-expressing neurons

**Figure 3:**
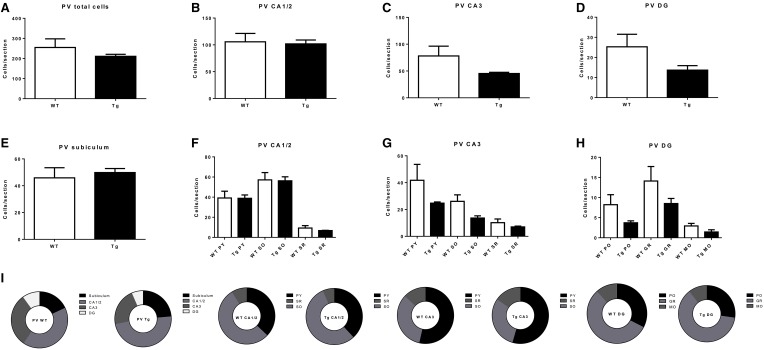
**Quantification of parvalbumin- (PV)-immunoreactive (IR) cells in the overall hippocampus (A), CA1 and CA2 (B), CA3 (C), dentate gyrus (DG; D) and subiculum (E), and in subregions within CA1 and CA2 (F), CA3 (G), and DG (H)**. **(I)** subregional proportion of PV-IR cells within hippocampal regions, for wild-type (WT) and transgenic (Tg) animals. PY, pyramidal layer; SO, stratum oriens; SR, stratum radiatum; MO, molecular layer; PO, polymorphic layer; GR, granule cell layer. WT, wild-type; Tg, transgenic.

**Figure 4:**
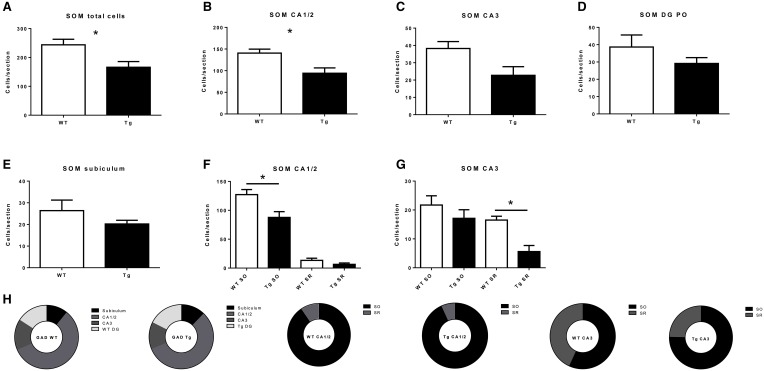
**Quantification of somatostatin- (SOM)-immunoreactive (IR) cells in the overall hippocampus (A), CA1 and CA2 (B), CA3 (C), dentate gyrus (DG; D) and subiculum (E), and in subregions within CA1 and CA2 (F), and CA3 (G)**. **(H)** subregional proportion of SOM-IR cells within hippocampal regions, for wild-type (WT) and transgenic (Tg) animals. SO, stratum oriens; SR, stratum radiatum; PO, polymorphic layer. WT, wild-type; Tg, transgenic. ^*^p < 0.05.

PV- and SOM-IR cells were observed in CA1/2, CA3, DG and subiculum in 6 month-old TgCRND8 mice (Figure 1; relative proportions are shown in Figures 3I, 4H, respectively), and their distribution was similar to that seen in anatomically matched hippocampal sections of the WT littermates.

PV-IR neurons in the hippocampus did not differ by genotype, either overall (*p* = 0.37; Figure 3A) or in CA1/2 (*p* = 0.84; Figure 3B) or CA3 (*p* = 0.21; Figure 3C), DG (*p* = 0.15; Figure 3D), or subiculum (*p* = 0.65; Figure 3E). Examining hippocampal subregions also revealed a lack of effect in CA1/2 pyramidal layer (*p* = 0.97), stratum oriens (*p* = 0.91), and stratum radiatum (*p* = 0.39; Figure 3F), CA1/3 pyramidal layer (*p* = 0.29), stratum oriens (*p* = 0.07), and stratum radiatum (*p* = 0.33; Figure 3G), and DG polymorphic layer (*p* = 0.15), granule cell layer (*p* = 0.22), and molecular layer (*p* = 0.15; Figure 3H).

Regarding SOM-IR cells, a significant decrease was observed both overall (*p* = 0.049; Figure 4A) and in CA1/2 (*p* = 0.040; Figure 4B). CA3 (*p* = 0.074; Figure 4C), DG (*p* = 0.29; Figure 4D), and subiculum (*p* = 0.30; Figure 4E) were not significantly affected. Concerning the layers of the CA regions, a significant decrease was observed in CA1/2 stratum oriens (*p* = 0.041; Figure 4F) and the CA3 stratum radiatum (*p* = 0.012; Figure 4G), but not CA1/2 stratum radiatum (*p* = 0.19) or CA3 stratum oriens (*p* = 0.35).

## Discussion

The principal finding of this study is that different hippocampal GABAergic neurons display remarkably distinct regional and sub-regional vulnerability to Aβ accumulation. Notably, in 6 month-old TgCRND8 mice, NPY-expressing GABAergic neurons are significantly decreased in all studied hippocampal regions except the subiculum. Regarding sub-regions, stratum oriens in CA is generally affected overall, as significantly fewer NPY neurons were found both in CA1/2 and CA3. Interestingly, SOM-expressing neurons of the stratum oriens were also found to be more vulnerable globally than in other layers of CA1/2, suggesting that this layer is a particular locus of AD-related GABAergic vulnerability. In addition, in the CA3 stratum radiatum layer, TgCRND8 showed fewer SOM- but not PV- or NPY-expressing neurons, suggesting that vulnerability in this region is neuronal subtype-specific to SOM-expressing neurons. Finally, overall hippocampal PV-expressing neurons demonstrated less vulnerability to the TgCRND8 AD model than other GABAergic neurons. The reasons for this apparent resistance of PV- compared to NPY- and SOM-expressing GABArgic neurons remain unknown. An interesting hypothesis is that PV-expressing neurons in the studied hippocampal sub-regions may be less active than NPY- and SOM-expressing neurons. This hypothesis will likely become testable in the near future, for example by combining electrophysiology and optogentics to functionally distinguish between these subtypes of GABAergic neurons. Importantly, recent studies have pointed also to a lower vulnerability of PV-expressing neurons in comparison to other types of GABAergic neurons. For instance, decrease in PV-expressing neurons occurs later and is less pronounced in the course of AD-like pathology than the loss of other types of GABAergic neurons (e.g., SOM- or calretinin-expressing neurons) in both olfactory bulb (Saiz-Sanchez et al., 2013) and olfactory cortex (Saiz-Sanchez et al., 2012) of the APPxPS1 mice. Similarly, PV-expressing neurons in the frontal cortex of APP/PS1 knock-in mice bearing double mutation in APP and PS1 display a relative resistance to Aβ accumulation; in spite of a 34% decrease in the total number of neurons between 10 and 2 month-old mice, the number of PV-expressing neurons was unchanged between the two ages (Lemmens et al., 2011). Moreover, in post-mortem brains of AD patients, PV-expressing neurons were found to be increased in the piriform cortex, whereas SOM- and calretinin GABAergic neurons were decreased (Saiz-Sanchez et al., 2014). Altogether, these data support relative resistance of PV-expressing GABAergic neurons to AD pathology.

These findings confirm and further extend our previous data on the significant loss of GABAergic neurons in CA regions of 6 month-old TgCRND8 AD-modeling mice (Krantic et al., 2012). They are also in agreement with the previous study by Ramos et al. (2006) concerning the sub-types of the most affected GABAergic neurons. This previous study has reported that NPY and SOM neurons are the most vulnerable in stratum oriens- lacunosum moleculare in CA regions of APP/PS1 at 6 months of age. In addition, we found a significant decrease of NPY neurons in dentate gyrus of TgCRND8 mice, similar to the loss reported in hilar performant path-associated cells (Ramos et al., 2006). However, in contrast to the APP/PS1 mice (Ramos et al., 2006), the decrease of NPY-IR cells contributes significantly to the observed loss of neurons in pyramidal layer of the CA1/CA2 region.

Regarding SOM neurons in stratum oriens, the loss we observed in 6 month-old TgCRND8 mice likely has a major impact in terms of GABAergic control of neuronal excitability, as all neurons of stratum oriens are GABAergic (Freund and Buzsaki, 1996). This is in line with what has been previously suggested for APP/PS1 mice (Ramos et al., 2006). However, in contrast to this study, we did not observe a significant decrease of SOM neurons in the dentate gyrus of TgCRND8 mice. In addition to SOM-expressing neurons, PV-expressing neurons were also unaffected in the DG, as were all studied GABA-expressing neuronal types in the subiculum. To our knowledge, this is the first such investigation of the subiculum, as this hippocampal sub-region has not been studied previously in terms of GABAergic neuronal vulnerability to Aβ. The relative resistance of the studied GABAergic neurons in the subiculum of 6 month-old TgCRND8 mice is intriguing. Indeed, the subiculum contains endogenous generator of hippocampal oscillatory activity (Goutagny et al., 2009; Jackson et al., 2014) which have been shown to be altered from the earliest stages of AD pathology in TgCRND8 mice (Goutagny et al., 2013).

Regarding PV neurons, our data differ from those provided by a previous study that pointed to a significant loss of these neurons in the CA1/2 region of APP/PS1 mice (Takahashi et al., 2010). The difference between our studies is likely due to the different age of the mice: 6 months for TgCRND8 and 10 months for APP/PS1 mice. It is less likely that the differences between our studies may come from the use of distinct mice strains bearing different mutations, as the pattern of the studied GABAergic neurons in the two transgenic AD mouse models appears globally similar. For instance, NPY neurons in the stratum oriens of both APP/PS1 (Ramos et al., 2006) and TgCRND8 (present study) strains are vulnerable to Aβ as compared to the non-transgenic animals.

The primary question raised by the observed alterations in the composition of the hippocampal population of the GABAergic neurons is how this functionally impacts neuronal excitability. In this light, the loss of SOM and NPY neurons in the stratum oriens of the CA1 region likely has major consequences in terms of control of pyramidal cell excitability. Indeed, cells in stratum oriens project to distal dendrites of CA1 pyramidal cells that provide dendritic inhibition (Maccaferri and Lacaille, 2003). As a corollary, dendritic inhibition may be reduced due to the loss of GABAergic neurons in the stratum oriens of the CA1 region with a subsequent dysregulation of entorhinal cortex input to CA1. Interestingly, a similar loss of SOM interneurons without alteration of PV neurons in the stratum oriens of CA1 has been reported in aged rat hippocampus and related to age-associated seizures and cognitive impairments (Stanley et al., 2012).

In conclusion, it is currently clear that the control of neuronal excitability through the control of GABAergic inhibitory input should be considered a therapeutic option for AD. In agreement, a number of studies using anti-epileptic drugs, likely acting through potentiation of GABAergic neurotransmission such as levetiracetam or topiramate, have reported beneficial effects on neuropathology and cognitive impairments in mouse AD models (Sanchez et al., 2012; Devi and Ohno, 2013; Shi et al., 2013; Zhang et al., 2014). Most importantly, levetiracetam has proven efficient in the management of not only spontaneous epileptiform activity, which accompanies the early stages of AD (Vossel et al., 2013), but also in improving cognition (Bakker et al., 2012). These exciting recent advances should foster further clinical assays for shared therapeutic options based on the use of anti-epileptic drugs to halt AD-associated cognitive impairment.

### Conflict of interest statement

The authors declare that the research was conducted in the absence of any commercial or financial relationships that could be construed as a potential conflict of interest.

## Abbreviations

Aβ: Amyloid beta
AD: Alzheimer's disease
CA: Cornu ammonis
DG: dentate gyrus
GABA: γ-aminobutyric acid
GR: granule cell layer
MO: molecular layer
NPY: neuropeptide Y
PO: polymorphic layer
PV: parvalbumin
PY: pyramidal layer
SO: stratum oriens
SOM: somatostatin
SR: stratum radiatum.
